# A Novel First Aid Stretcher for Immobilization and Transportation of Spine Injured Patients

**DOI:** 10.1371/journal.pone.0039544

**Published:** 2012-07-09

**Authors:** Yan-Sheng Liu, Ya-Ping Feng, Jia-Xin Xie, Zhuo-Jing Luo, Cai-Hong Shen, Fang Niu, Jian Zou, Shao-Feng Tang, Jiang Hao, Jia-Xiang Xu, Li-Ping Xiao, Xiao-Ming Xu, Hui Zhu

**Affiliations:** 1 People’s Liberation Army Clinical Center for Spinal Cord Injury, Kunming General Hospital of People’s Liberation Army, Kunming, P. R. China; 2 Department of Orthopaedics, The First Affiliated Hospital of The Fourth Military Medical University, Xi’an, P.R. China; 3 Department of Clinical Laboratory Science,The First Wuxi Affiliated Hospital of Nanjing Medical University, Wuxi, P.R. China; 4 Emergency Department, Kunming General Hospital of People’s Liberation Army, Kunming, P. R. China; 5 Yunnan Emergency Center, Kunming, P. R. China; 6 Spinal Cord and Brain Injury Research Group, Department of Neurological Surgery, Stark Neurosciences Research Institute, School of Medicine, Indiana University, Indianapolis, Indiana, United States of America; Hertie Institute for Clinical Brain Research, University of Tuebingen., Germany

## Abstract

Effective immobilization and transportation are vital to the life-saving acute medical care needed when treating critically injured people. However, the most common types of stretchers used today are wrought with problems that can lead to further medical complications, difficulty in employment and rescue, and ineffective transitions to hospital treatment. Here we report a novel first aid stretcher called the “emergency carpet”, which solves these problems with a unique design for spine injured patients. Polyurethane composite material, obtained by a novel process of manually mixing isocyanate and additives, can be poured into a specially designed fabric bag and allowed to harden to form a rigid human-shaped stretcher. The effectiveness of the emergency carpet was examined in the pre-hospital management of victims with spinal fractures. Additionally, it was tested on flat ground and complex terrain as well as in the sea and air. We demonstrated that the emergency carpet can be assembled and solidified on the scene in 5 minutes, providing effective immobilization to the entire injured body. With the protection of the emergency carpet, none of the 20 patients, who were finally confirmed to have spinal column fracture or dislocation, had any neurological deterioration during transportation. Furthermore, the carpet can be handled and transported by multiple means under differing conditions, without compromising immobilization. Finally, the emergency carpet allows the critically injured patient to receive multiple examinations such as X-ray, CT, and MRI without being removed from the carpet. Our results demonstrate that the emergency carpet has ideal capabilities for immobilization, extrication, and transportation of the spine injured patients. Compared with other stretchers, it allows for better mobility, effective immobilization, remarkable conformity to the body, and various means for transportation. The emergency carpet is promising for its intrinsic advantages in the pre-hospital management of accident victims.

## Introduction

Worldwide, physical trauma is a leading cause of death and disability. Data from the World Health Organization (Global Burden of Diseases, Injuries, and Risk Factors Study 2005) showed about 16,000 people died from injury every day, and for each person who died, several thousand more were injured, many of them with permanent sequelae. Injury accounts for 16% of the global burden of disease. Injury care and management have seen significant advances over the last decade. Together with evolving new therapies, efforts should be made to improve strategies for emergency management. Since most injuries take place away from medical facilities, the need for rapid, valid, and effective immobilization and transportation of the critically wounded is urgent for emergency providers in accidents or military actions. Effective immobilization becomes particularly crucial when wounds and injuries of the spinal column and cord occur.

The first aid stretcher and its equivalents have played an important life-saving role in these emergency circumstances. Deficiencies in the conventional stretcher and its equivalents, such as scant flexibility and insufficient immobilization for the wounded, lead to additional injury or aggravation of the primary lesion [1]. Therefore, it has become urgent to invent a more efficient and practical medical tool to meet the requirements of various emergency circumstances. Improvements in these emergency situations may prevent further damage to the spinal cord during transport to emergency medical facilities, resulting in a decrease in the proportion of complete spinal injuries.

The purpose of this study was to design and configure a novel first aid stretcher with the following characteristics: (1) It should be simple to manage and convenient to carry. (2) It should provide effective immobilization, while being easily transported by different methods (such as pushing, dragging, hanging, floating, etc) according to the situation, without exacerbating the severity of the injury due to ineffective or incomplete immobilization. (3) It should be environmentally compatible, water repellent, flame retardant, and X-ray permeable. We have developed such a new first aid stretcher – the “emergency carpet”, and tested it in patients with spine injuries, in different topographical and emergency conditions to evaluate its efficacy.

## Materials and Methods

A camouflage colored refractory cotton fabric bag was shaped to resemble the outline of a human body, with elongated flaps on both sides of the forehead and neck region that were designed to immobilize the head and neck. Belts were set up around the bag for binding and handling. The bag can easily be folded and compacted for carrying. Polyurethane composite materials were chosen to be the filler, which was obtained by a novel foaming-curing-molding process of polyurethane rigid foam. The isocyanate and special additives are contained separately in two ring-pull/hand pull-tab cans(Can A–-polyurethane curing agent(800 ml):isocyanate 45 wt%; Can B–-additives(200 ml): composite polymer polyol 43 wt%; silicon oil 3.5 wt%, catalyst 2.5 wt%, inorganic additives 6 wt%.). These materials can be packaged into a compact musette bag along with the fabric bag that will become the stretcher, a roller, and a stick.

The process is implemented by mixing the polyurethane curing agent with additives, and stirring manually with a stick at normal temperature and pressure for one minute, to get a polyurethane precursor solution. Then the composite solution is poured into a human-shaped fabric bag. After the above steps, rollers are used to planish the fabric bag in order to make sure that the mixture is uniformly distributed into each part of the bag. As soon as the carpet is ready, the critically injured individual can be placed upon it and then the carpet is wrapped around the body using the attached belts. After wrapping it snuggly around the patient, the stretcher adapts to each patient’s unique body shape and solidifies in 5 minutes.

All research involving human subjects was approved by the Science and Research Committee of Kunming General Hospital of PLA. Since the first aid stretcher was always used at the rescue scene when the victims were in critical conditions, the written patient consent forms were not requested. However, patient verbal consent for the use of the emergency carpet was solicited by emergency medical personnel. In collaboration with the local Urgent Care Center (UCC), Yunnan Emergency Center, application of the emergency carpet on victims with spinal fractures was performed in 2009. Ambulances were equipped with several sets of the emergency carpet, and the emergency medical personnel were trained to proficiently evaluate the state of the spinal trauma. Once the injured patient was evaluated and highly suspected to have injury of the vertebral column without neurologic symptoms as defined by the American Spinal Injury Association (ASIA) score system [2], the emergency carpet was employed to immobilize and transport the victim. After arriving at the hospital, an X-ray film or CT scan was taken, without removing patients from the emergency carpet, to confirm the spinal column fracture or dislocation. Neurological evaluation was conducted on victims who were confirmed to have spinal column fracture or dislocation in order to evaluate whether neurological deterioration occurred during the transportation procedure. After utilizing the emergency carpet for 3 months, 18 emergency care providers completed a survey about their experiences. The survey asked about the validity of the emergency carpet as well as any possible negative effects. The efficacy of the emergency carpet was examined on flat ground, complex terrain, at sea, and in the air.

## Results

### Formation of the Emergency Carpet

**Figure 1:**
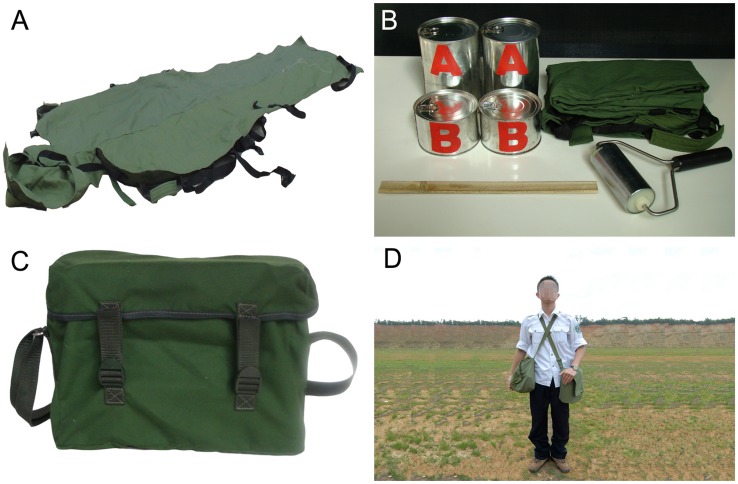
Components of the emergency carpet musette bag. A: The human-shaped fabric bag. B: Material cans (two sets), fabric bag, roller, and stick. C: Musette bag containing the whole set of emergency carpet. D: Emergency provider shouldering two musette bags easily.

**Figure 2:**
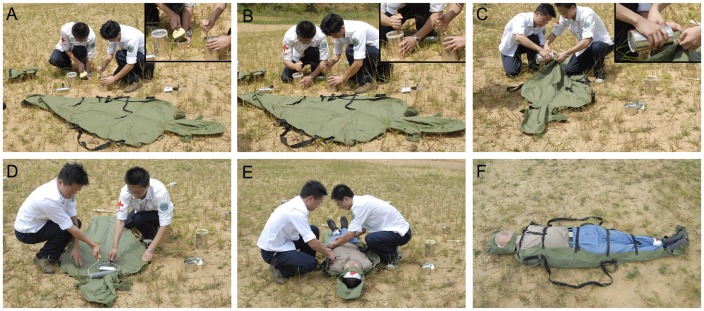
Formation process of the emergency carpet. A: Mixing material of two cans. B: Stirring manually with a stick. C: Pouring the composite solution into human-shaped fabric bag. D: Rolling with the rollers. E: Wrapping the injured person on the carpet. F: Solidified carpet with the injured person.

The whole set of the emergency carpet can be packaged in a carrying bag weighing around 4 kg (Fig.1). After mixing and pouring the special polyurethane composite material into the fabric bag and conducting the binding and molding procedure, a rigid emergency carpet, completely molded in conformity to the body is created within 5 minutes (Fig. 2).

### Application of the Emergency Carpet

#### Pre-hospital application

**Figure 3:**
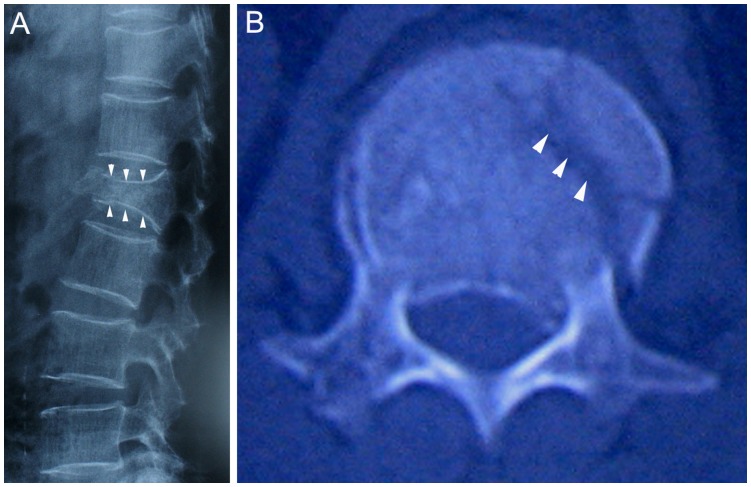
Spinal column fracture confirmed by X-ray and CT. A: Compression fracture of lumbar vertebra confirmed by X-ray (lateral view, arrows). B: Compression fracture of lumbar vertebra confirmed by CT (arrows).

From August to December 2009, 25 victims were immobilized and transported by the local Urgent Care Center utilizing the emergency carpet. Twenty of these patients were finally confirmed to have spinal column fracture or dislocation by X-ray or CT (Fig.3), and were hospitalized for internal fixation (Table 1). None of the 20 patients had any neurological symptoms or deterioration from the time of injury to discharge date as evidenced by a consistent ASIA score throughout the period of medical management.

**Table 1:** Patients of vertebral column injuries transported with the emergency carpet.

Number	ID	Gender	Age	Injury Date	Injured Vertebra
1	10381706	M	34	2009.08.02	C2
2	10382852	F	37	2009.08.11	T12, L1
3	10383208	M	38	2009.08.17	L2
4	10383572	M	62	2009.09.01	T9, 12, L2
5	10384520	M	46	2009.09.04	T12
6	10388924	F	46	2009.09.15	L2
7	10389600	M	36	2009.09.20	L1
8	10390524	F	27	2009.10.05	T12, L3
9	10391082	F	16	2009.10.12	L1, 2
10	10391433	M	44	2009.10.15	L3
11	10391521	F	38	2009.10.16	T11
12	10391768	M	48	2009.10.19	T12
13	10392149	M	47	2009.10.23	L2, 3
14	10392916	M	39	2009.11.02	L3
15	10393012	F	34	2009.11.04	C4
16	10393566	M	28	2009.11.09	L1, 3
17	10395208	M	35	2009.11.24	L1
18	10395483	M	56	2009.11.29	L2
19	10396154	M	56	2009.12.06	L1
20	10396943	M	37	2009.12.10	T11

Survey results indicate that participating emergency care providers (n = 18) did not feel any discomfort when mixing the reagents, nor did they feel any burning sensations while in contact with the formed emergency carpet (Table 2). All but one participating emergency care provider rated the mixing of reagents to be an easy process. When emergency care providers were asked if the emergency carpet was safe and valid to put a patient with spine injuries onto for long-distance transportation, all but one, who abstained from answering, responded positively. There were no reports or complaints of discomfort from patients being transported by the emergency carpet. All participating emergency care providers reported no fracture, necrosis, or skin burns in any patient transported by the emergency carpet. The survey questions and results are summarized in Table 2.

**Table 2:** Summary of survey results collected from emergency care providers.

Survey questions	Yes	No	Abstained
1. Did you feel any discomfort in your nose or eyesduring mixing the reagents for the emergency carpet?	0/18(0%)	18/18(100%)	0/18(0%)
2. Did you feel any heat or burning while in contact with theemergency carpet during or after the forming procedure?	0/18(0%)	18/18(100%)	0/18(0%)
3. Was the forming procedure of the emergency carpet difficultfor you to manage?	1/18(6%)	17/18(94%)	0/18(0%)
4. Do you feel that it is safe or valid to put a patient with spine injuries on the emergencycarpet for long-distance transportation?	17/18(94%)	0/18(0%)	1/18(6%)
5. Have you ever heard any complaints of discomfort from the emergency carpetby the patients after being placed on the carpet?	1/18(6%)	17/18(94%)	0/18(0%)
6. Have you found any necrosis or burns on the skin of the patients in contactwith the emergency carpet?	0/18(0%)	17/18(94%)	1/18(6%)
7. During transportation in different situations, have you found any fracture of patientsusing the emergency carpet?	0/18(0%)	18/18(100%)	0/18(0%)

#### Application on flat ground

**Figure 4:**
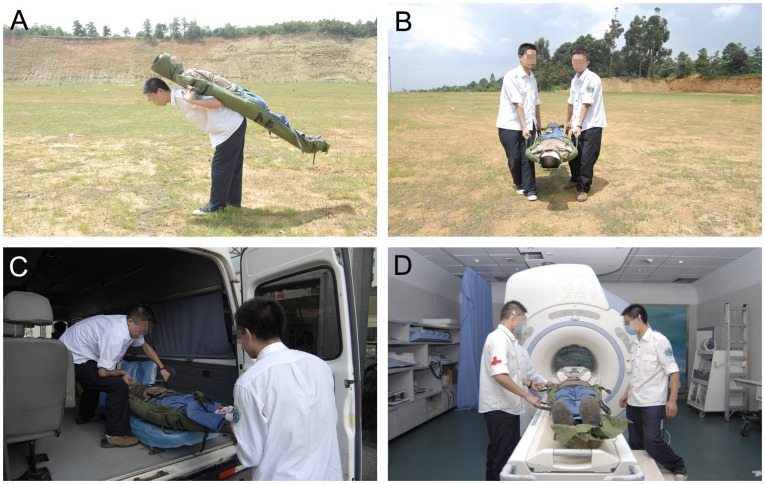
Applications of the emergency carpet on flat ground. A: Carrying on the back by one man. B: Carrying by two men. C: Placing the victim in the ambulance. D: Compatible with MRI scan.

In most situations the terrain was simple and did not require the assistance of specialized rescue teams. Therefore, the emergency providers assembled the emergency carpet for the critically injured person. After wrapping the injured patient within the carpet, emergency care providers either carried the emergency carpet by the fabric belt handles, or placed the patient within the emergency carpet on a medical truckle. Since the carpet is X-ray permeable and metal free, the X-ray film, CT scan, or MRI scan can be performed without moving the patient out of the emergency carpet, which has the significant advantage of not causing secondary damage to the spinal cord (Fig.4).

#### Application on complex terrain

**Figure 5:**
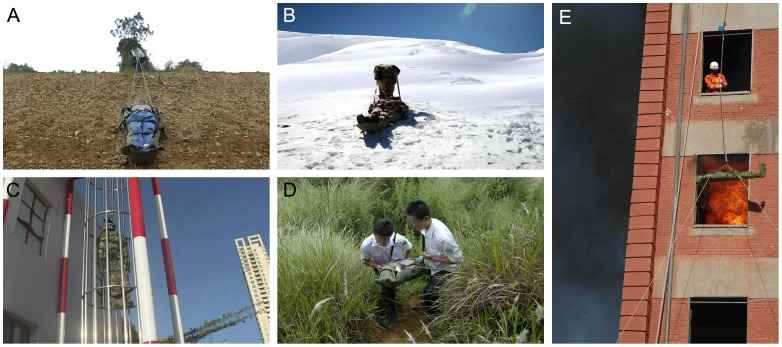
Applications of the emergency carpet on complex terrain. A: Transportation on a steep slope. B: Sliding as a sleigh. C: Upright transportation within a pipeline. D: Shouldering by two men in a mountain area. E: Suspension in the high-rise building.

When rescue operations were carried out under unconventional conditions, the emergency carpet was utilized as a versatile first aid stretcher fulfilling different needs (Fig.5). The earthquake rescue workers could transport the injured victims over long distances, such as uneven terrain in a mountainous area, with the emergency carpet. During the fire accident, which took place in a high-rise building, fire crews could quickly immobilize victims who were in a coma, severely burned, or suffering from spinal fracture using the emergency carpet, and evacuate the victim to a more secure area utilizing the suspension rescuing system. Even on a snow field, the emergency carpet can be dragged as a sleigh with the injured person onboard.

#### Application under special conditions

**Figure 6:**
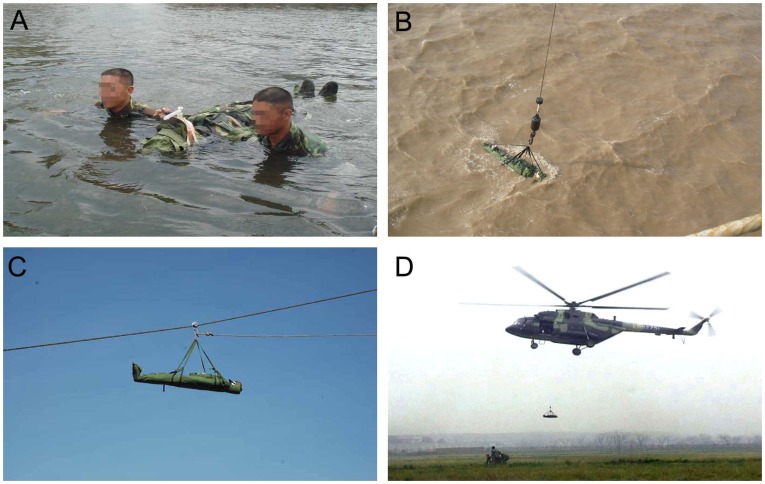
Applications of the emergency carpet under special conditions. A: Floating in water. B: Transporting by wave compensation crane. C: Transporting by suspension system between ships. D: Transporting by helicopter.

The emergency carpet can be handled not only on land, but also in water, or in the air (Fig.6). In a maritime environment, wounded persons can be transported directly in water or by wave compensation crane, as well as by suspension system between ships. If needed, helicopters can directly overhang the emergency carpet in order to rescue the wounded.

## Discussion

Novel modification of ingredients allowed us to create the first foaming-curing-molding process for the polyurethane rigid foam. This process is completed by stirring the mixture of isocyanate and additives manually with a stick, at normal temperature and pressure. Utilizing this patented technique, a novel stretcher, i.e. the emergency carpet, with the aforementioned characteristics was created.

Though the forming process of the emergency carpet involves a series of chemical reactions, the main components are mostly macromolecular polyether polyol and polyurethane pre-polymers, making the material non-corrosive and non-flammable. During the foaming process, water vapor was generated as the foaming gas, and a slight increase of the reagents’ temperature (0.7°C) was found. The reagents are non-toxic substances and are presented as viscous liquid before mixing, which become stickier after mixing. The emergency care providers can wear gloves to prevent hand stickiness at the time of reagent mixing. Since the reagents and the mixing products are non-toxic, they are harmless to both emergency care providers and patients. Hardness of the emergency carpet was determined by its weight-bearing capacity. The current version of the emergency carpet was designed with a weight-bearing capacity of 90 Kg. Under such bearing capacity, no fracture of the emergency carpet was observed under temperatures between −20°C and 60°C.

The initial management of an emergency medical situation must include scrupulous consideration for the fixation and transportation of the critically injured person. It is important not to aggravate the primary injury, nor to cause or exacerbate a neurological injury. [3]. First aid stretchers and the equivalents have played an important role in the history of emergency management. There are various types of stretchers available in current pre-hospital practice. Spinal boards, the military stretcher, vacuum mattress, and the Life Support for Trauma and Transport platform (LSTAT) are the typical representatives which have been highly acclaimed and widely used in emergency events and military actions. There are advantages and disadvantages to each of these stretcher types.

Spinal board or backboard is the most common tool for emergency management, especially for the spinally injured person, and it has long been thought to be the best surface for the spinally injured patient. As reported in much research, the curved nature of the spine means that spinal boards focus most of the weight and pressure of the body on the sacral and thoracic kyphosis. Spinal boards give little support to other areas, and the lumbar lordosis receives no support. Healthy subjects could experience severe pain lying on a hard spinal board, and this might confuse the diagnosis or exclude actual injury identification [4]. The pressures exerted on these areas of the body while on the spinal board are enormous and damaging. It is possible for this flat backboard to lead to pressure sores in those who have sustained injury to the spinal cord [5], [6]. The military stretcher was designed to optimize stability and extrication ability, adapting to the battlefield environment. Despite its collapsable structure, the folding pole canvas stretcher is still insufficiently mobile. Moreover, Lovell found high interface pressures on this type of stretcher that might lead to a higher risk of pressure necrosis [7].

The vacuum mattress is a flexible sack of polystyrene beads that becomes rigid on the application of a vacuum. It was proven to provide significantly superior stability and be considerably more comfortable than a backboard, as well as dramatically reduce sacral interface pressures from the potentially ischemic levels generated with the backboard [8]–[11]. However, the vacuum mattress provides insufficient immobilization for the head and neck. Moreover, it is wider than most ambulance trolleys and therefore not compatible with some vehicles. It is also bulky to store when not in use, which is a great drawback to its extrication ability. When encountering a rescue environment with rough, rocky surfaces that may puncture the sack, a loss of vacuum would render the vacuum mattress useless.

The LSTAT is a stretcher-based miniature intensive care unit designed by the United States Army, which provides appropriate equipment to detect and manage critical events in patient care. The platform functions as a mobile ICU and has been preliminarily tested with success in combat settings [12], [13]. With multiple integrated systems (ventilator, defibrillator, suction, hemodynamic monitors, infusion and invasive monitoring channels, capnography, blood analysis, and electrocardiography), the LSTAT becomes heavy and complicated for medical staff to handle and use appropriately. As a complex mobile ICU, the platform should be equipped for a rescue team with vehicles rather than carried by medical personnel to a rough terrain rescue.

### Conclusion

Application of the emergency carpet in different scenarios revealed that the emergency carpet has ideally integrated abilities for immobilization, extrication, and transportation of the critically injured patient, especially those with spine injuries. Firstly, it possesses great mobility. We have invented a novel first aid stretcher with superb mobility which is remarkably compact prior to use, that can be transformed from a soft small material into a rigid full size stretcher. The musette bag contains the entire contents to create the carpet that can be easily carried by medical personnel. Each emergency provider can easily shoulder 4 bags without influencing the mobility of their upper limbs, indicating uneaqualed advantage in mobility compared with other stretchers. Secondly, the emergency carpet can be molded simply in a short time by two people. It requires fewer people and less training to assemble the emergency carpet than the vacuum mattress and the LSTAT. Thirdly, since the emergency carpet is completely molded in conformity to the body, the pressures distribute uniformly on the surface, decreasing the risk of pressure necrosis while providing at least the same comfort as the vacuum mattress. Moreover, while the rigid foam countered perfectly to the body provides effective immobilization to the injured part of the body, especially to the injured spine, the emergency carpet also possesses ideal extrication ability without affecting immobilization. Fourthly, the carpet integrates functions of many military stretchers, such as the desert stretcher, jungle stretcher, snowfield stretcher, and the Neil Robertson stretcher, resulting in continuous transportation of the wounded during wartime, which is one of the important goals of integrated military health services. Since the carpet can be transported by different means while carrying the patient, adapting to various terrains including mountainous areas, high-rise buildings, and even water, it can be used effectively in a wide variety of emergency circumstances. This is particularly true in the mountain rescue environment where it is impossible for a helicopter to land. In such a situation, the immobilized injured person can be directly suspended and transferred into the rescue helicopter while within the emergency carpet. Lastly, due to the X-ray-permeable and metal-free characteristics of the polyurethane composite material, it is convenient for the critically injured person to undergo X-ray, CT, or MRI examinations while wrapped within the carpet, thus avoiding extra injuries caused by the shifting procedures within the hospital, as well as reducing obstructions during the transportation process. In addition, the camouflage properties and fire resistance have made application of the emergency carpet more effective. We feel that the new emergency carpet has great potential in emergency and critical care medicine.
